# Lin^−^ PU.1^dim^GATA‐1^−^ defines haematopoietic stem cells with long‐term multilineage reconstitution activity

**DOI:** 10.1111/cpr.13490

**Published:** 2023-05-05

**Authors:** Haoyu Xu, Shaojing Tan, Yu Zhao, Lin Zhang, Weiyun Cao, Xing Li, Jiayi Tian, Xiaojing Wang, Xiaoyan Li, Fengchao Wang, Jiani Cao, Tongbiao Zhao

**Affiliations:** ^1^ State Key Laboratory of Stem Cell and Reproductive Biology, Institute for Stem Cell and Regeneration, Institute of Zoology Chinese Academy of Sciences Beijing China; ^2^ Beijing Institute for Stem Cell and Regenerative Medicine Beijing China; ^3^ University of Chinese Academy of Sciences Beijing China; ^4^ National Institute of Biological Sciences (NIBS) Beijing China

## Abstract

Despite extensive characterization of the state and function of haematopoietic stem cells (HSCs), the use of transcription factors to define the HSC population is still limited. We show here that the HSC population in mouse bone marrow can be defined by the distinct expression levels of *Spi1* and *Gata1.* By using a double fluorescence knock‐in mouse model, PGdKI, in which the expression levels of PU.1 and GATA‐1 are indicated by the expression of GFP and mCherry, respectively, we uncover that the HSCs with lymphoid and myeloid repopulating activity are specifically enriched in a Lin^−^PU.1^dim^GATA‐1^−^ (LPG) cell subset. In vivo competitive repopulation assays demonstrate that bone marrow cells gated by LPG exhibit haematopoietic reconstitution activity which is comparable to that of classical Lin^−^Sca1^+^c‐kit^+^ (LSK). The integrated analysis of single‐cell RNA sequence data from LPG and LSK‐gated cells reveals that a transcriptional network governed by core TFs contributes to regulation of HSC multipotency. These discoveries provide new clues for HSC characterization and functional study.

## Graphical abstract

Haematopoietic stem cells (HSC) population in mouse bone marrow can be defined by the distinct expression levels of *Spi1* and *Gata1.* By using a double fluorescence knock‐in mouse model, PGdKI, in which the expression levels of PU.1 and GATA‐1 are indicated by the expression of GFP and mCherry respectively, we uncover that the HSCs with lymphoid and myeloid repopulating activity are specifically enriched in a Lin^−^PU.1^dim^GATA‐1^−^ (LPG) cell subset. In vivo competitive repopulation assays demonstrate that bone marrow cells gated by LPG exhibit haematopoietic reconstitution activity which is comparable to that of classical Lin^−^Sca1^+^c‐kit^+^ (LSK). The integrated analysis of single‐cell RNA sequence data from LPG and LSK gated cells reveals that a transcriptional network governed by core TFs contributes to regulation of HSC multipotency.

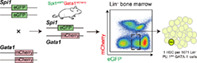

## 1 INTRODUCTION

Haematopoiesis is a hierarchical and dynamic process involving haematopoietic cells at distinct differentiation stages like multipotent stem cells, lineage‐restricted progenitors and specialized blood cells. The isolation and identification of functional haematopoietic stem cells (HSCs) is a major challenge due to their rarity and heterogeneity. An early study showed that HSCs existing in a subpopulation called Lin^−^Sca1^+^c‐kit^+^ (LSK), which is negative for markers of mature blood cells and positive for Sca1 and c‐kit (LSK), have the ability to reconstitute all blood cell types in mouse bone marrow.^1^ Subsequently, a series of surface markers have been combined with LSK for purification and characterization of HSCs. For example, it has been proved that 40% of CD34^–/dim^LSK, 47% of CD150^+^CD48^−^LSK, and 43% of CD150^+^CD48^−^CD45^+^EPCR^+^ cell populations can achieve long‐term multi‐lineage reconstitution at single‐cell levels.^2^, ^3^, ^4^

The surface marker EPCR, encoded by the *Procr* gene, has been suggested to track the maturation process from pre‐HSCs to HSCs in mouse embryos.^5^ Cells purified solely based on EPCR expression show potent haematopoietic reconstitution activity, which is comparable to the cells gated by the Hoechst 33342 staining method in mouse bone marrow.^6^, ^7^ Similarly, the expression of platelet integrin CD41 (αIIb), a surface marker regulated by GATA‐1, successfully marks the transient initiation of definitive haematopoiesis during murine ontogeny.^8^ CD41 is expressed in a subset of long‐term myeloid‐biassed CD150^+^CD48^−^LSK cells in adult mouse bone marrow, and the expression of CD41 in adult long‐term HSCs becomes prevalent with age.^9^

Given the success in using surface markers to gate HSC populations, it is still hard to precisely define HSC subsets by using surface markers only; furthermore, the surface markers of HSCs may differ among species.^10^, ^11^, ^12^, ^13^ Accumulating evidence suggests that haematopoietic transcription factors (TFs) play pivotal roles in the maintenance and differentiation of HSCs. For example, GATA‐2, Myb, c‐Myc and HOX regulate HSC stemness and are responsible for HSC commitment.^14^, ^15^, ^16^, ^17^, ^18^ Lineage‐specific TFs play essential roles in controlling the progression of haematopoietic differentiation, and their coordinated expression primes the developmental potential of cells with mixed‐lineage states. For example, TFs IRF8 and GFI1 initiate the specialization of granulocyte‐monocyte progenitors (GMPs) to granulocytes and monocytes, while KLF1 and FLI1 mediate erythroid versus megakaryocyte fate decisions in HSCs.^19^, ^20^, ^21^ PU.1 and GATA‐1 proteins directly bind to each other as a negative regulator of a network of downstream genes.^22^, ^23^ They are cross‐antagonistic transcription factors involved in erythroid and myeloid cell lineage priming in HSCs.^24^

Currently, some TFs involved in haematopoietic cell fate determination are used to identify HSCs.^25^ For example, lineage tracing studies have revealed that certain TFs, such as MECOM, GATA‐2, Hox‐B4, Hox‐B5, TAL‐1, TCF‐15 and TIE‐2, are specifically expressed in mouse HSCs.^26^, ^27^, ^28^, ^29^, ^30^, ^31^, ^32^ These TFs play essential roles in maintaining HSC self‐renewal and have been used to label HSCs.^33^, ^34^, ^35^, ^36^, ^37^ However, without evaluation of the long‐term haematopoietic capacity, many bone marrow cells expressing those TFs have been mislabelled as HSCs.^25^ In addition, platelet‐related TFs such as GATA‐1, TPO‐R and vWF are highly expressed in long‐term HSCs and down‐regulated in short‐term HSCs.^38^ While vWF^+^ HSCs can give rise to myeloid‐biassed HSCs and lymphoid‐biassed HSCs, a specific population of these cells, called platelet‐biassed HSCs, also effectively and stably replenish megakaryocyte/platelet‐lineage cells but not other blood cell lineages.^39^, ^40^ While vWF^+^ HSCs are highly enriched in the megakaryocyte niche, NG2^+^ arteriolar niche cells selectively maintain vWF^−^ HSCs in the bone marrow.^30^, ^41^ These observations suggest that both multipotent HSCs and platelet‐biassed HSCs are regulated by distinct niches. Understanding the transcriptional network maintaining HSC multipotency is important for precise identification and characterization of the functional HSCs.

In this study, we have demonstrated that HSCs express TFs PU.1 and GATA‐1 at distinct levels, and multipotent HSCs are specifically enriched in the Lin^−^PU.1^dim^GATA‐1^−^ (LPG) subset of mouse bone marrow cells. By scRNA‐seq analysis on the LPG and LSK populations, we reveal that a gene regulatory network, controlled by core TFs, plays pivotal roles in HSC multipotency maintenance. Our discovery provides new clues for understanding transcriptional regulation of mouse HSCs.

## 2 MATERIALS AND METHODS

### 2.1 Mice

All animal experiments were approved by the Ethics Committee in the Institute of Zoology, Chinese Academy of Sciences in accordance with the Guidelines for Care and Use of Laboratory Animals established by the Beijing Association for Laboratory Animal Science. C57BL/6 mice, including CD45.1, CD45.2 and CD45.1/CD45.2, congenic mice, at the age of 8–12 weeks were used for transplantation assays. The Spi1‐IRES‐puroR‐p2A‐eGFP (C57BL/6 background) and Gata1‐IRES‐puroR‐p2A‐mCherry (C57BL/6 background) knock‐in mice were bred at the Transgenic Research Center, National Institute of Biological Sciences (Beijing). The C57BL/6‐*Hoxb5* ^*em2(2A‐tdTomato)Smoc*^ mice were purchased from Shanghai Model Organisms Center, Inc. (Shanghai), and C57BL/6 mice were purchased from Vital River Laboratory Animal Technology Co. Ltd. (Beijing) and SPF Biotechnology Co., Ltd. (Beijing) at 7–10 weeks of age.

### 2.2 Generation of Spi1^eGFP^ ^/eGFP^ and Gata1^mCherry^ ^/mCherry^ double knock‐in (PGdKI) mice

IRES‐puroR‐p2A‐eGFP and IRES‐puroR‐p2A‐mCherry expression cassettes were inserted after the stop codons of the *Spi1* and *Gata1* genes, respectively, by homologous recombination. Briefly, the guide RNA sequence for the *Spi1* gene (CACCTACCAGTTCAGCGGCG) or the *Gata1* gene (GTTGTAGGCGATCCCAGCAG) was designed according to the CRISPR design tool (http://crispr.mit.edu/) and inserted into the BbsI‐digested pX330 plasmid (Addgene, Cambridge, MA; plasmid 42230). The plasmid sequence was confirmed. A donor template for homologous recombination was constructed containing the sequences on each side of the stop codon of *Spi1* (Gene ID: 20375) or *Gata1* (Gene ID: 14460). Both upstream and downstream arm sequences were amplified by the polymerase chain reaction (PCR) from C57BL/6 mouse genomic DNA and cloned into the pBluescript II SK(+) vector adjacent to the insertion fragment IRES‐puroR‐p2A‐eGFP or IRES‐puroR‐p2A‐mCherry. Using the pX330 plasmids and the donor templates, the knock‐in mice were generated by CRISPR‐Cas9‐mediated gene‐editing technology.^42^ Offspring were then genotyped using PCR analysis with the primer set for *Spi1* knock‐in mice (upstream arm forward primer: TCTCTGCCATCCCTCACTGACCTTC and IRES reverse primer: GCACACCGGCCTTATTCCAAGC; EGFP forward primer: ACATGGTCCTGCTGGAGTTCGTG and downstream arm reverse primer: TGCTATGCTTATCTCCGAGTCGTCCAG; forward primer on the upstream arm: GGAGGGTCCCCATAAAATC and reverse primer on the downstream arm: TGATCCCTGAGCCCTGATA) and the primer set for the *Gata1* knock‐in mice (upstream arm forward primer: CAAACGGGCAGGCACCCAATG and IRES reverse primer: GCACACCGGCCTTATTCCAAGC; mCherry forward primer: CGAGGACTACACCATCGTGGAACAG and downstream arm reverse primer: TCTGCCTTGCCTCTGCCACCG; forward primer on the upstream arm: TCCCTCTTTGCTCCTCTTTCT and reverse primer on the downstream arm: CTCCATGCTCCACTTGACACT). These primers can detect both wild‐type (WT) and knock‐in alleles at the same time. The Spi1‐IRES‐puroR‐p2A‐eGFP and Gata1‐IRES‐puroR‐p2A‐mCherry double knock‐in mice (C57BL/6 background) were obtained by mating the two types of knock‐in mice together. The homozygous mice were utilized in this study.

### 2.3 Flow cytometry and cell sorting

Flow cytometry and cell sorting were performed on a fluorescence‐activated cell sorting (FACS) Aria II cell sorter (BD Biosciences) and data were analysed using FlowJo_v10. Bone marrow cells were isolated by flushing mouse femurs with phosphate‐buffered saline (PBS) and passing the cells through 40‐μm strainers before analysis and sorting. The single‐cell suspensions were incubated in cell staining buffer (Biolegend, 420,201) with TruStain FcX™ PLUS antibody (Biolegend, 156,603) to block Fc‐receptors for 10 min, and then incubated with the specific antibody for 30 min on ice. The fluorescently‐labelled antibodies against cell surface markers were used in the relevant cell staining for HSC enrichment as follows: anti‐Lineage – APC (BD Pharmingen, 558,074), anti‐Ly‐6A/E(Sca1) – PerCP‐Cyanine5.5 (eBioscience, 45–5981‐82), anti‐CD117(c‐kit) – APC/Cy7 (Biolegend, 105,826), anti‐CD34 – eFluor 450 (eBioscience, 48–0341‐80), anti‐CD127 – eFluor 660 (eBioscience, 50–1271‐80), anti‐CD16/32 – Brilliant Violet 421 (Biolegend, 101,332), anti‐CD34 – Brilliant Violet 421 (Biolegend, 152,208), anti‐CD135 – Brilliant Violet 421 (Biolegend, 135,314), anti‐CD150 – Brilliant Violet 421 (Biolegend, 115,926), anti‐CD41 – Brilliant Violet 510 (Biolegend, 133,914), anti‐CD48 – Brilliant Violet 510 (Biolegend, 103,443).

### 2.4 CFUs assay

A total of 12,500 total bone marrow cells were isolated from the legs and hips of PGdKI mice and cultured in methylcellulose‐based medium (StemCell technology, 03434) with recombinant cytokines (including EPO) for mouse cells. Number and morphology of the colonies were analysed with phase contrast microscope after 7 days of culture.

### 2.5 Competitive repopulation assay

Recipient mice were treated with lethal irradiation (9.0 Gy) for bone marrow transplantation experiments. 10^5^ Lin^−^ bone marrow cells (CD45.2) were sorted using a flow cytometer (BD FACS Aria II) depending on the expression of PU‐1 and GATA‐1, and mixed with 5 × 10^5^ whole bone marrow competitor cells (CD45.1) in 200 μL PBS, and injected into tail veins of recipient mice. For secondary transplants, 2 × 10^6^ whole bone marrow cells from primary recipient mice were transplanted into lethally irradiated recipient mice.

### 2.6 Limiting dilution assay

A series of dilutions (300, 900 and 2700) of the donor cells (CD45.2) were competed against 2 × 10^5^ whole bone marrow competitor cells (CD45.1) as above. At 16 weeks after transplantation, HSC frequency was calculated by peripheral blood analysis from the Extreme Limiting Dilution Analysis website (http://bioinf.wehi.edu.au/software/elda/).^43^ Then, the frequency of HSCs (competitive repopulating units, CRUs) was estimated.

### 2.7 Peripheral blood analysis

Sixteen weeks after transplantation, peripheral blood was collected from the tail vein of recipient mice. Collection was repeated at 4‐week intervals. Then, the erythrocytes were lysed using erythrocyte lysis buffer (Shanghai Yeasen Biotech Co. Ltd), and the remaining cells were stained with antibodies against the following markers: anti‐CD3 – PerCP/Cyanine5.5 (Biolegend, 100,218), anti‐CD45R/B220 – APC (Biolegend, 103,212), anti‐CD11b – Brilliant Violet 421 (Biolegend, 101,236), anti‐Ly‐6G/Ly‐6C (Gr‐1) – Brilliant Violet 421 (Biolegend, 108,434), anti‐CD45.2 – Brilliant Violet 605 (Biolegend, 109,841), anti‐CD45.1 – Brilliant Violet 785 (Biolegend, 110,743).

### 2.8 Immunofluorescence microscopy

Sorted cells were harvested and fixed with 4% paraformaldehyde, washed with Dulbecco's PBS (Corning Inc., R21‐031‐CV), permeabilized using 0.2% Triton‐X100 (Sigma Aldrich, X100) for 30 min, blocked with 2% bovine serum albumin (Sigma Aldrich, A1933) for 1 h, stained with appropriate primary antibodies overnight at 4°C, and incubated with secondary antibodies for 2 h at room temperature. The cell nuclei were counterstained with DAPI (4′,6‐diamidino‐2‐phenylindole). The images were captured from frozen sections by Zeiss LSM880 microscopy.

### 2.9 Western blotting

Cells were ice‐lysed in RIPA buffer (50 mM Tris–HCl, pH 7.4, 150 mM NaCl, 0.5% sodium deoxycholate, 1% Nonidet P‐40, 5 mM EGTA, 2 mM EDTA, 10 mM NaF) for 30 min. Protease inhibitor cocktail (04693116001, Roche, Mannheim, Germany) and 1 mM PMSF (ST506, Beyotime, Shanghai, China) were added. The lysates were mixed with loading buffers, boiled, centrifuged and subjected to SDS‐PAGE electrophoresis prior to transfer to PVDF membranes (Millipore). After blocking with 5% non‐fat milk in TBST (50 mM Tris–HCl pH 8.0, 150 mM NaCl, 0.1% Tween‐20), the membrane was incubated with anti‐GATA1 (a640847, Abcam) and anti‐β‐actin (ab8226, Abcam). The secondary antibody was IRDye® 800CW goat anti‐mouse IgG secondary antibody. Finally, protein expression was detected by Odyssey® CLx Infrared Imaging System.

### 2.10 Single‐cell RNA sequencing

Single‐cell RNA sequencing (scRNA‐seq) was performed in cooperation with CapitalBio Technology Inc. (Beijing, China). Briefly, bone marrow tissues were flushed from adult mouse femurs with PBS buffer and passed through 40 μm strainers, and the LSK cells were collected and counted using a flow cytometer (BD FACS Aria II). Then single‐cell RNA‐seq libraries were constructed according to the instructions accompanying the single cell 3′ Library and Gel Bead Kit V3 (10× Genomics, 1,000,075). The cDNA libraries were subsequently generated, amplified, and assessed for quality control using the Agilent 4200, and single‐cell RNA sequencing was further performed on the Illumina Novaseq6000 sequencer. Read pre‐processing was performed using the 10 × Genomics workflow. For quality control, we set the threshold and removed cells with more than 10% of reads mapping to mitochondrial genes (regarded as low‐quality cells that exhibit extensive mitochondrial contamination). A final dataset of 12,814 LSK cells was preserved to map the landscape of HSC development.

LPG cells were enriched and processed as described above. ScRNA‐seq data of 12,814 LSK cells merged with 19,608 LPG cells were computed and visualized using R packages.

### 2.11 Cell clustering and analysis

For clustering, cells were divided into subsets and reclustered for optimal resolution based on the number of cells according to Seurat (version = 4.0.6) and pySCENIC (version = 0.11.2) tutorials.^44^, ^45^, ^46^

For the Seurat analysis, we used the Read10× function to read the output from the 10× Genomics Cell Ranger (version = 4.0) mapped to the mm10 reference genome. This returned a unique molecular recognition (UMI) count matrix, which was used to create a Seurat object. For quality control, we visualized quality control metrics, and applied them to filter cells. We removed cells with more than 30,000 reads and fewer than 3000 reads, or over 10% mitochondrial reads. Next, we normalized the data using the NormalizeData function in Seurat. We selected the top 2000 variably expressed genes (VEGs) utilizing the FindVariableFeatures function in Seurat with the default setting. The VEGs were further scaled by the ScaleData function in Seurat. For principal component analysis (PCA) and Uniform Manifold Approximation and Projection (UMAP), the first 20 principal components were performed and then used. To identify clusters of cells by a shared nearest neighbour (SNN) modularity optimization, we utilized the FindNeighbors and FindClusters functions. To visualize and explore these datasets., we used the RunUMAP function to run nonlinear dimensional reduction. To identify the DEGs among clusters, we used the FindMarkers function in Seurat.

In addition, we used pySCENIC to infer the gene regulatory networks (GRNs) based on DNA motif analysis and the co‐expression of TFs and target genes. TFs were identified using GENIE3/GRNBoost and compiled into modules (regulons) that were further subjected to cis‐regulatory motif analysis by RcisTarget. To identify cell states and their regulators, we assessed the network activity in each individual cell (AUCell), and scoring regulons in the cells. We identified stable cell states based on their gene regulatory network activity (cell clustering).

### 2.12 Statistical analyses

Statistical details and number of replicates are shown in the corresponding figure legends. Analyses were performed using Prism 8 (GraphPad Software). Statistical significance was calculated using unpaired *t* tests between the indicated groups, *p*‐values <0.05 were considered as statistically significant. *p*‐Values are indicated by asterisks as follows: *p < 0.05, **p < 0.01, ***p < 0.001.

### 2.13 Data and code availability

The raw data files for the scRNA sequencing data have been deposited in the Science Data Bank. All relevant data supporting the findings of this study are also available from the lead contact (tbzhao@ioz.ac.cn) upon request.

## 3 RESULTS

### 3.1 ScRNA‐seq analysis of LSK cells reveals that multipotent HSCs and erythroid‐biassed HSCs can be distinguished by expression of *Spi1* and *Gata1*

**FIGURE 1:**
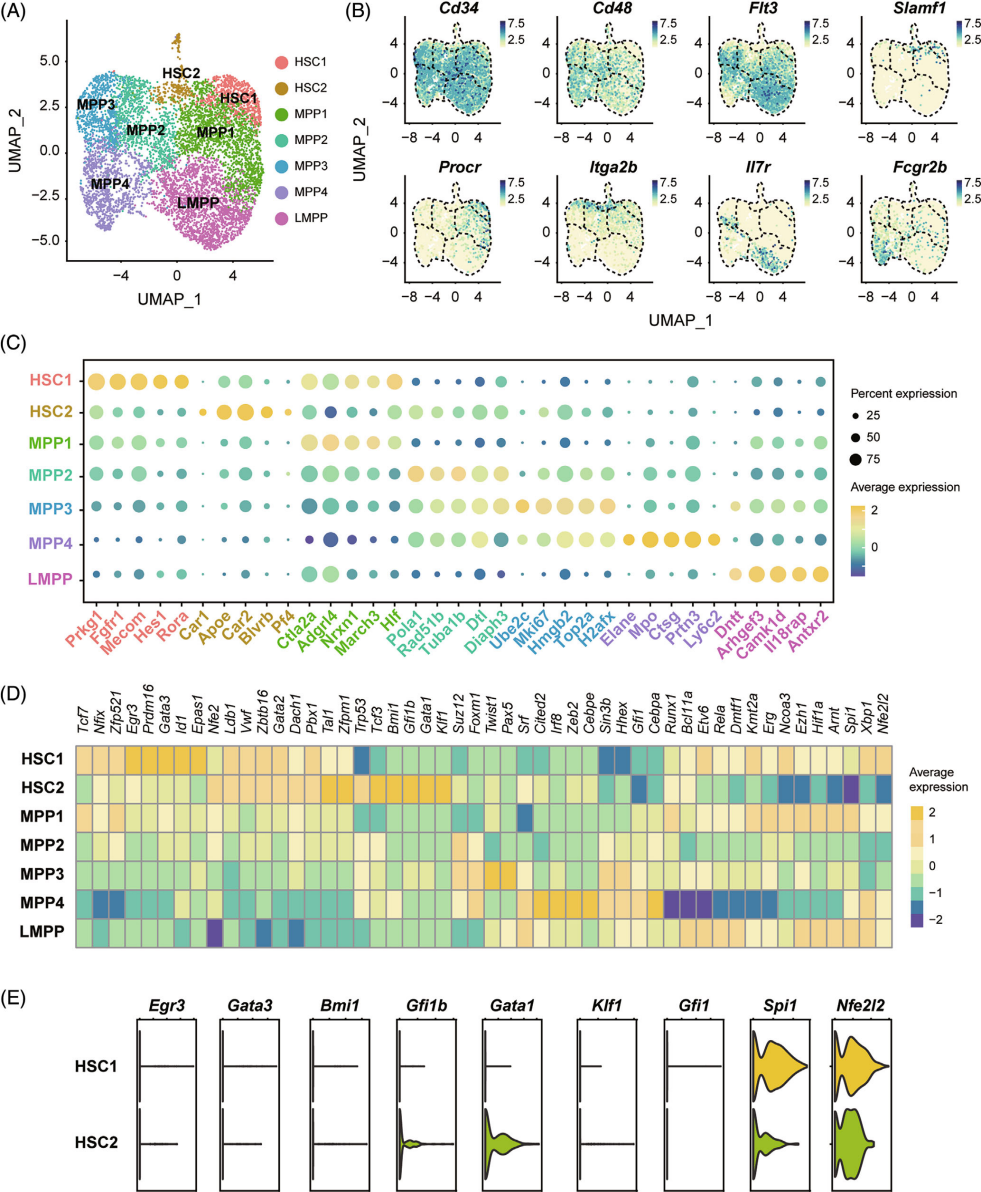
Single‐cell RNA sequencing analysis of the Lin^−^Sca1^+^c‐kit^+^ (LSK) cellular population in mouse bone marrow. (A) Uniform Manifold Approximation and Projection (UMAP) showing 7 clusters identified from 10 × scRNA‐seq analysis of all mouse bone marrow LSK cells. Each dot represents one cell. (B) The expression pattern of eight HSPC‐related cell marker genes within the cells from A. (C) Dot plot showing the average expression levels and expression proportions of the top 5 different expression genes (DEGs) within each cluster. The size of the dot represents the proportion of cells expressing the indicated gene within a cluster, and the colour of the dot indicates the average expression level of cells within a cluster. The top 5 DEGs are labelled in the same font colour as the corresponding cluster in A. (D) Heatmap showing the mean expression of haematopoietic lineage‐specific transcription factors in each cluster. (E) Violin plot showing the expression of transcription factor genes, including *Spi1* and *Gata1*, in the HSC1 and HSC2 clusters. HSC, haematopoietic stem cell; HSPC, haematopoietic stem and progenitor cell; LMPP: lymphoid‐primed multipotent progenitor cell; MPP, multipotent progenitor cell.

To explore the potential TFs that can be used to isolate and characterize haematopoietic stem cells (HSCs) in adult mouse bone marrow, we first performed single‐cell RNA sequencing (scRNA‐seq) on LSK cells. Seven subpopulations of LSK cells, including HSC1, HSC2, multipotent progenitors 1 (MPP1), MPP2, MPP3, MPP4 and lymphoid‐primed multipotent progenitors (LMPP), were clustered and identified according to the expression of six traditional HSC surface markers—*Cd34*, *Cd48*, *Flt3* (encoding CD135), *Slamf1* (encoding CD150), *Procr* (encoding EPCR), and *Itga2b* (encoding CD41)—and two progenitor cell surface markers, *Il7r* (encoding CD127) and *Fcgr2b* (encoding CD32) (Figure 1A,B).^44^ Clusters HSC1 and HSC2 both showed specific expression of *Slamf1* and limited expression of *Cd34*, *Cd48* and *Flt3* compared to the other subpopulations of LSK cells, indicating their HSC characteristics (Figure 1B). The distinct expression patterns of *Procr* and *Itga2b* between clusters HSC1 and HSC2 suggests that the HSC population in LSK cells is composed of discrete subsets with varied potential.

We then analysed the differentially expressed genes (DEGs) among these clusters (Figure 1C). The top 5 DEGs in the HSC1 cluster are *Prkg1*, *Fgfr1*, *Mecom*, *Hes1* and *Rora*, among which *Fgfr1*, *Mecom* and *Hes1* have been shown to be essential for HSC mobilization and functioning.^29^, ^47^, ^48^ In the HSC2 cluster, there is high expression of two genes involved in HSC proliferation (*Apoe* and *Pf4*) and megakaryocytic function, and three erythroid‐related genes (*Car1*, *Car2* and *Blvrb*), which indicates the erythroid‐biassed differentiation potency of this cluster.^49^, ^50^, ^51^, ^52^, ^53^

Two TF genes, *Hlf* and *Ctla2a*, which promote haematopoietic transplantation engraftment, are highly expressed in the MPP1 cluster.^54^, ^55^ The gene *Diaph3*, which is expressed in erythroid progenitor cells,^56^ is highly expressed in both the MPP2 and MPP3 clusters. In the MPP3 cluster, there is strong expression of the gene *Hmgb2*, which has been shown as a regulator for HSC regeneration,^57^ and *Mki67*, which encodes a marker of proliferation.^58^ The expression of the monocyte‐related genes *Elane*, *Ctsg*, *Prtn3* and *Ly6c2* and the neutrophil‐related gene *Mpo* are raised in MPP4, indicating the myeloid differentiation trajectory of this cluster.^59^, ^60^, ^61^ The expression levels of the lymphoid‐related genes *Dntt* and *Il18rap* are increased in the LMPP cluster, which is consistent with the lymphoid‐primed characteristics of these cells^62^, ^63^ (Figure 1C).

There are 3 broad trajectories of HSC differentiation landscape: lymphoid, myeloid (granulocyte and monocyte), and erythroid (erythrocyte and megakaryocyte).^64^ We next assessed the expression of haematopoietic TFs in the seven clusters to predict their differentiation trajectories (Figure 1D). The results showed that both the HSC1 and HSC2 clusters strongly expressed *Ldb1*, *Zbtb16*, *Gata2* and *Pbx1*, which are essential for the maintenance of HSCs.^15^, ^65^, ^66^, ^67^ Unexpectedly, we found enhanced expression of the platelet‐related gene *Vwf* in HSC1 and HSC2.^41^ In addition, the HSC1 cluster strongly expressed the myeloid‐ and lymphoid‐related genes *Egr3* and *Gata3*.^68^, ^69^ In the HSC2 cluster, the expression levels of the erythroid trajectory‐specific TF genes *Bmi1*, *Gfi1b*, *Gata1* and *Klf1* ^70^, ^71^, ^72^, ^73^ were enhanced, while the expression levels of the myeloid‐ and lymphoid‐related genes *Gfi1*, *Spi1* (encoding PU.1) and *Nfe2l2* ^74^, ^75^, ^76^ were reduced (Figure 1D). These results indicate that the HSC1 cluster possesses myeloid and lymphoid differentiation potential, whereas the HSC2 cluster possesses erythroid‐megakaryocyte differentiation potential, which is consistent with the results from DEG analysis (Figure 1C).

Given the distinct expression patterns of *Spi1* and *Gata1* in haematopoietic lineages and their reciprocal activation effects on HSC specification, we aimed to evaluate whether they can be used together to distinguish the multipotent HSC populations. We found that the two putative HSC clusters in LSK cells can be distinguished by the expression differences between *Spi1* and *Gata1* (Figure 1E).

### 3.2 Haematopoietic immunophenotype of PU.1^eGFP^GATA‐1^mCherry^ (PGdKI) reporter mice

**FIGURE 2:**
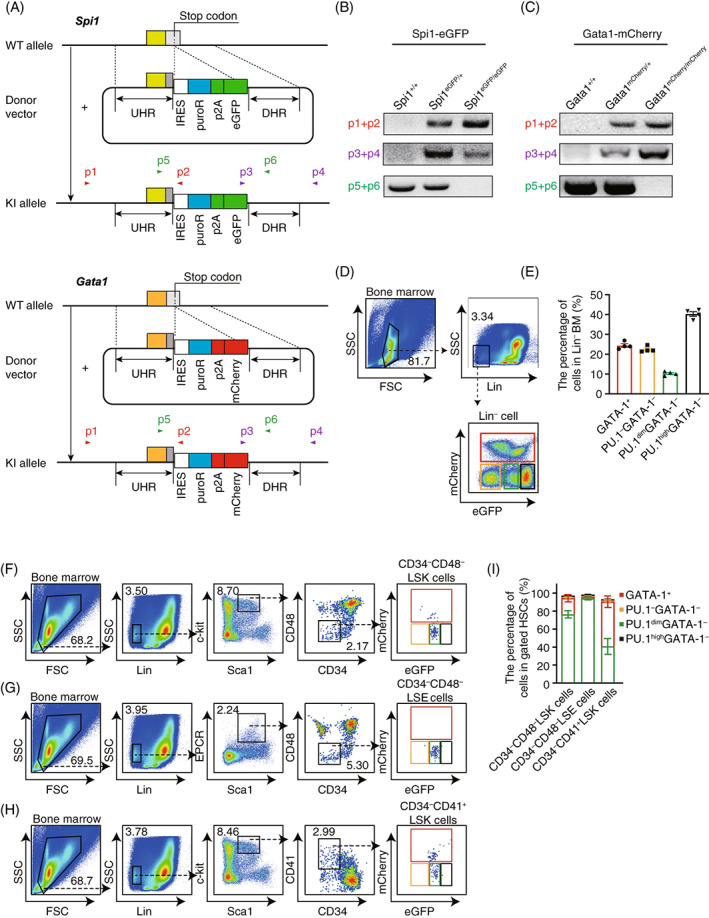
*Spi1* is moderately expressed in haematopoietic stem cell (HSC) populations gated by traditional surface markers. (A) Diagram of the knock‐in alleles in the mouse genes *Spi1* and *Gata1*. IRES‐puroR‐p2A‐eGFP and IRES‐puroR‐p2A‐mCherry expression cassettes were inserted after the stop codons of *Spi1* and *Gata1*, respectively. The locations of primers for genotyping analysis are indicated. (B–C) Genotyping analysis of the knock‐in mice. The primers used are indicated in A. (D) FACS analysis shows that the Lin^−^ bone marrow cells of PGdKI mice are classified into four cell subsets according to different expression levels of eGFP and mCherry. (E) The percentages of the four indicated subpopulations in Lin^−^ bone marrow cells. Data are shown as mean ± SEM, *n* = 4. (F–I) Expression of PU.1 and GATA‐1 in HSCs identified by traditional HSC surface markers: (F) Lin^−^Sca‐1^+^c‐kit^+^ (LSK) CD34^–/low^CD48^−^, (G) Lin^−^Sca‐1^+^EPCR^+^ (LSE) CD34^–/low^CD48^−^, and (H) LSKCD34^–/low^CD41^+^ cells. (I) The contribution of the indicated cell subsets in the CD34^−/dim^CD48^−^LSK population, the CD34 ^−/dim^CD48^−^LSE population, and the CD34^−/dim^CD41^+^LSK population (mean ± SEM, *n* = 3). GATA‐1^+^ cells are gated in the red box, PU.1^−^GATA‐1^−^ cells are gated in the orange box, PU.1^dim^GATA‐1^−^ cells are gated in the green box, and PU.1^high^GATA‐1^−^ cells are gated in the black box.

We generated *Spi1* ^eGFP/eGFP^ *Gata1* ^mCherry/mCherry^ double knock‐in (PGdKI) mice, in which the IRES‐puroR‐p2A‐eGFP and IRES‐puroR‐p2A‐mCherry expression cassettes were inserted immediately after the stop codons of *Spi1* and *Gata1*, respectively (Figure 2A). The genotype of *Spi1* ^eGFP/eGFP^ and *Gata1* ^mCherry/mCherry^ knock‐in mice was verified by PCR analysis (Figure 2B,C). PGdKI mice were generated by cross‐breeding of *Spi1* ^eGFP/eGFP^ and *Gata1* ^mCherry/mCherry^ mice.

We next evaluated the expression of GATA‐1 and PU.1 in distinct haematopoietic lineages from PGdKI mice. Existing studies have shown GATA‐1 expression in myeloid cells and PU.1 expression in lymphoid cells.^77^ GATA‐1 is strongly expressed in megakaryocytic‐erythroid precursors (MEPs) and its expression decreases during differentiation into T, B, Mφ, neutrophil and platelet cells.^19^, ^78^, ^79^, ^80^ PU.1 expression occurs in CLP, GMP, B, DC, Mφ and neutrophil cells.^81^, ^82^, ^83^, ^84^, ^85^, ^86^, ^87^, ^88^, ^89^, ^90^, ^91^, ^92^ Accordingly, the expression of mCherry (which reports GATA‐1 expression) is high in MEPs and declines in B, T, platelet, neutrophil and Mφ cells; and eGFP (which reports PU.1 expression) is expressed in common lymphoid progenitor (CLP), GMP, B, Mφ and neutrophil cells at distinct levels and is highly expressed in a subset of dendritic cells (DCs) (Figure S1A–D).

### 3.3 PU.1 is moderately expressed in HSCs gated by canonical haematopoietic surface markers

To test the feasibility of using the distinct expression levels of PU.1 and GATA‐1 to gate and characterize HSCs, we analysed bone marrow cells of PGdKI mice by a FACS. The lineage‐depleted (Lin^−^) bone marrow cells of PGdKI mice were successfully divided into 4 independent subpopulations—mCherry^+^, eGFP^−^mCherry^−^, eGFP^dim^mCherry ^−^, and eGFP^high^mCherry^−^—according to the fluorescence intensity of GFP and mCherry (Figure 2D). Then, we took the eGFP^−^, eGFP^dim^ and eGFP^high^ cells which were FACS‐sorted depending on GFP intensity, and we stained them with anti‐GFP and anti‐PU.1 antibodies and observed them by immunofluorescence microscopy. The results indicated that the expression of PU.1 corresponded precisely with eGFP expression (Figure S2A). In addition, the results of western blotting showed that the protein level of GATA‐1 is dramatically higher in sorted mCherry^+^ cells compared with mCherry^−^ cells, indicating that the mCherry expression can be used to monitor GATA‐1 expression (Figure S2B,C). Thus, the mCherry^+^, eGFP^−^mCherry^−^, eGFP^dim^mCherry^−^ and eGFP^high^mCherry^−^ populations were denoted as GATA‐1^+^, PU.1^−^GATA‐1^−^, PU.1^dim^GATA‐1^−^ and PU.1^high^GATA‐1^−^, and they account for 24.35% ± 0.91%, 22.40% ± 0.74%, 10.28% ± 0.51% and 40.28% ± 1.18% of Lin^−^ cells, respectively (Figure 2E).

We then examined whether PU.1 and/or GATA‐1 are expressed in HSCs defined by canonical cell surface markers (Figure 2F–I). The HSCs gated by CD34^−/dim^CD48^−^LSK contained 74.97% ± 3.28% PU.1^dim^GATA‐1^−^ and 17.90% ± 3.23% GATA‐1^+^ cells; these gated HSCs lacked the PU.1^−^GATA‐1^−^ and PU.1^high^GATA‐1^−^ subsets (Figure 2F,I). For HSCs gated by CD34^−/dim^CD48^−^Lin^−^Sca1^+^EPCR^+^ (CD34^−/dim^CD48^−^LSE; 92.53% ± 1.17%), the majority of cells were PU.1^dim^GATA‐1^−^, with background frequencies of GATA‐1^+^, PU.1^−^GATA‐1^−^ and PU.1^high^GATA‐1^−^ subsets (Figure 2G,I). In contrast, CD34^−/dim^CD41^+^LSK HSCs were composed of 39.47% ± 7.15% PU.1^dim^GATA‐1^−^ and 49.50% ± 5.27% GATA‐1^+^ cells, with limited PU.1^−^GATA‐1^−^ and PU.1^high^GATA‐1^−^ populations (Figure 2H,I). These data indicate that multipotent HSCs are available in PU.1^dim^GATA‐1^−^ subsets of Lin^−^ cells. We named this Lin^−^PU.1^dim^GATA‐1^−^ subpopulation as LPG.

### 3.4 The LPG population of mouse bone marrow cells enriched multipotent HSCs

**FIGURE 3:**
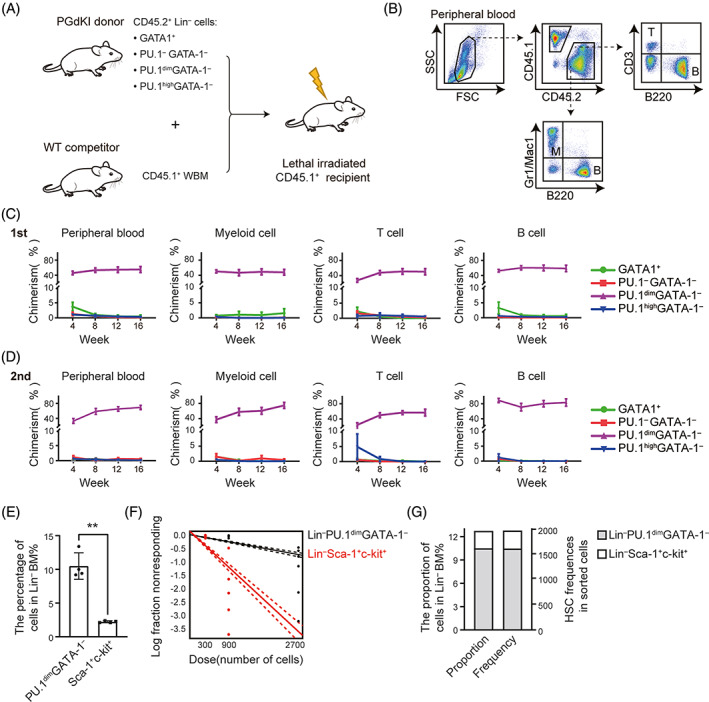
Lin^−^PU.1^dim^GATA‐1^−^ gates functional haematopoietic stem cells (HSCs) in mouse bone marrow cells. (A) Schematic of the competitive repopulation assay. Donor cells (GATA‐1^+^, PU.1^−^GATA‐1^−^, PU.1^dim^GATA‐1^−^ or PU.1^high^GATA‐1^−^) were sorted from Lin^−^ BM cells of PGdKI mice (CD45.2^+^) by FACS according to the expression level of eGFP and mCherry. For each group, the indicated CD45.2^+^ donor cells were mixed with WT CD45.1^+^ competitor cells and transplanted into lethally irradiated CD45.1^+^ recipient mice. WBM, whole bone marrow. (B) The gating strategy to identify CD45.2^+^ donor cells and CD45.1^+^ competitor cells in the peripheral blood of CD45.1^+^ murine recipients. T: CD3^+^ T cells; B: B220^+^ B cells; M: Gr1/Mac1^+^ myeloid cells. (C) The primary transplant of PU.1^dim^GATA‐1^−^, but not GATA‐1^+^, PU.1^−^GATA‐1^−^ and PU.1^high^GATA‐1^−^, results in haematopoietic repopulation in recipient mice. (D) The second transplantation assay indicates that the Lin^−^PU.1^dim^GATA‐1^−^ cells possess long‐term haematopoiesis ability (Data shown are average values from three independent experiments. Error bars denote SEM. *n* = 8 mice for GATA‐1^+^ subsets; *n* = 9 mice for PU.1^−^GATA‐1^−^ subsets; *n* = 8 mice for PU.1^dim^GATA‐1^−^ subsets; *n* = 6 mice for PU.1^high^GATA‐1^−^ subsets finally). (E) Statistical analysis of Sca1^+^c‐kit^+^ and PU.1^dim^GATA‐1^−^ cells in Lin^−^ bone marrow (*n* = 4 mice, mean ± SD). Two‐tailed paired Student's *t*‐test, **p < 0.01. (F) Enumeration of functional HSCs by ELDA (extreme limiting dilution analysis, *p* < 0.05) at 16 weeks post‐transplantation. Mice were transplanted with varying doses of Lin^−^Sca1^+^c‐kit^+^ cells (red, *n* = 3 mice for 300 and *n* = 6 mice for 900) and Lin^−^PU.1^dim^GATA‐1^−^ cells (black, *n* = 6 mice for 900 and *n* = 6 mice for 2700) isolated from bone marrow. Data are from 3 independent experiments. (G) The cell percentage ratio of Sca1^+^c‐kit^+^ and PU.1^dim^GATA‐1^−^ cells in Lin^−^ bone marrow (left, 2.23%: 10.5% = 0.21) is consistent with the HSC frequency ratio of LSK cells and LPG cells (right, 334:1671.2 = 0.20).

Next, we investigated the haematopoietic reconstitution abilities of Lin^−^GATA‐1^+^, Lin^−^PU.1^−^GATA‐1^−^, Lin^−^PU.1^dim^GATA‐1^−^ and Lin^−^PU.1^high^GATA‐1^−^ cells by bone marrow transplantation experiments. The GATA‐1^+^, PU.1^−^GATA‐1^−^, PU.1^dim^GATA‐1^−^, and PU.1^high^GATA‐1^−^ cells were sorted from Lin^−^ bone marrow cells of PGdKI mice (B6 CD45.2^+^), mixed with B6 CD45.1^+^ competitor bone marrow cells, and transplanted into B6 CD45.1^+^ recipient mice (Figure 3A). The chimeric proportions of T cells (CD45.2^+^CD3^+^), B cells (CD45.2^+^B220^+^), and myeloid cells (CD45.2^+^Gr1/Mac1^+^) in the recipient peripheral blood were examined by a FACS (Figure 3B). Interestingly, the PU.1^dim^GATA‐1^−^ subset, but not the GATA‐1^+^, PU.1^−^GATA‐1^−^ and PU.1^high^GATA‐1^−^ subsets, of Lin^−^ bone marrow cells were able to undergo long‐term lymphoid and myeloid lineage differentiation in irradiated recipient mice after the first and second bone marrow transplantation (Figure 3C,D). These data provide evidence that the multipotent HSCs are enriched in the LPG population of mouse bone marrow cells.

We then compared the haematopoietic reconstitution capacity between LSK and LPG cells. LPG cells account for about 50.53% ± 3.86% of LSK cells, while LSK cells occupy around 12.43% ± 1.76% of LPG cells (Figure S2E,F). To measure the frequency of functional HSCs, we performed limiting dilution assays on LSK and LPG cells, and the chimerism rates of peripheral blood cells in the recipients were examined 16 weeks after transplantation to evaluate the competitive repopulating units (CRUs). The results showed that the frequency of functional HSCs in LSK and LPG cells is 1/334 versus 1/1671.2 (Figure 3F,G). Notably, Sca1^+^c‐kit^+^ cells account for around 2.23% of Lin^−^ cells, while PU.1^dim^GATA‐1^−^ gated approximately 10.5% of Lin^−^ cells (Figure 3E,G). Thus, LPG gates the multipotent HSC population at the same level as LSK in mouse bone marrow.

### 3.5 Core TFs control the multipotency of HSCs via distinct gene regulatory networks

**FIGURE 4:**
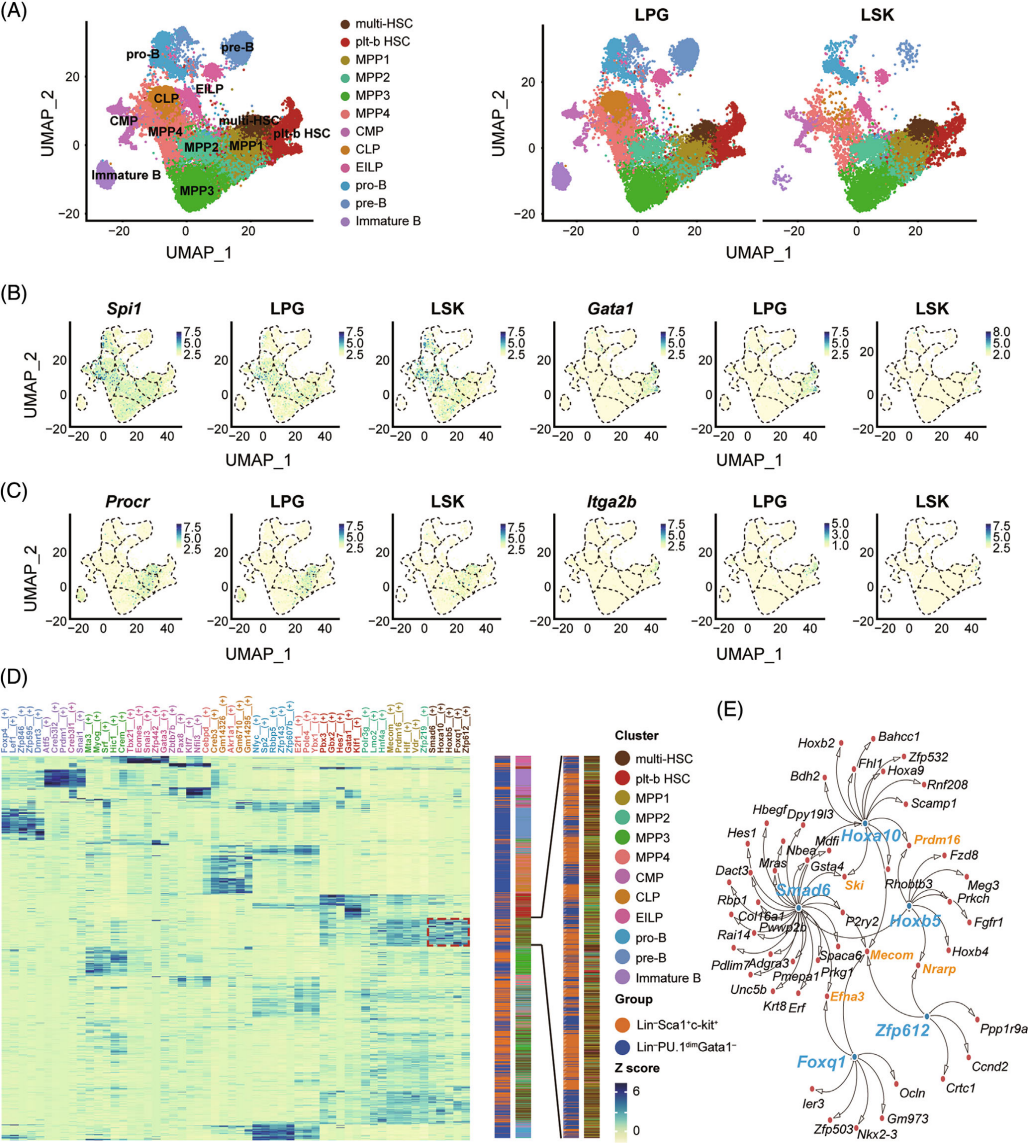
Integrated scRNA‐seq analysis of Lin^−^PU.1^dim^GATA‐1^−^ (LPG )and Lin^−^Sca1^+^c‐kit^+^ (LSK) cells reveal novel transcriptional regulation networks in haematopoietic stem cells (HSCs). (A) Uniform Manifold Approximation and Projection (UMAP) visualization of integrated single‐cell sequencing data from LSK cells and LPG cells. Each dot represents one cell. (B) The expression of *Spi1* and *Gata1* in distinct clusters of LPG and LSK cells. (C) The expression pattern of *Procr* and *Itga2b* in distinct clusters of LPG and LSK cells. (D) SCENIC results for the clustered cells. The clustered regulon activity matrix plots the top five active regulons within each distinct cluster and displays them as a heatmap of z‐scored enrichment values. The coloured bars on the right of the heatmap indicate the group and the cluster of every single cell. The putative regulons of HSCs are marked by a red dotted box and are magnified. (E) HSC‐related gene regulatory network. Blue dots indicate core TF genes and red dots indicate downstream genes. *Smad6*, *Hoxa10*, *Hoxb5*, *Foxq1* and *Zfp612* serve as master regulators for active regulons in multipotent HSCs. The co‐regulated downstream genes of master regulators are labelled in orange text, and other target genes which are regulated by only one master transcription factor are marked in black text. CLP, common lymphoid progenitor cells, CMP, common myeloid progenitor cells, EILP, early innate lymphoid progenitor cells; MPP, multipotent progenitor cells; multi‐HSCs: multipotent HSCs; plt‐b HSCs: platelet‐biassed HSCs.

We next performed the scRNA‐seq analysis on LPG cells, and integrated these data with the scRNA‐seq data from LSK cells by the “Harmony” method. Combined with canonical haematopoietic surface markers, the integrated LSK and LPG cells were designated into two HSC clusters and 10 progenitor clusters, which were visualized by UMAP^93^ (Figures 4A–C and S3). Similar to the HSC1 and HSC2 clusters identified in LSK cells, the HSC cluster expressing *Spi1* but not *Gata1* was denoted to be the multipotent HSC cluster (multi‐HSC), while the HSC cluster expressing *Gata1* was classified to be the platelet‐biassed HSC cluster (plt‐b HSC) (Figure 4B). The *Spi1* and *Gata1* expression patterns in the HSC clusters of LPG cells exhibited high similarity to their counterparts in LSK cells. The distinct expression pattern of *Procr* and *Itga2b* in the two HSC clusters further supported this designation (Figure 4C).

To gain insight into the mechanism underlying HSC multipotency regulation, we searched for the transcriptional signatures within each cluster by pySCENIC, in which the positive correlation coefficient of expression between a TF and its targets can be detected. The resultant top 5 active TFs were listed for each cluster (Figure S4A).^45^, ^94^ The TFs *Zfp612*, *Foxq1*, *Hoxb5*, *Hoxa10* and *Smad6* were identified at the top of the transcriptional activity list in the multi‐HSC population (Figure S4A). Interestingly, analysis of the clustered cells in a regulon activity matrix, which plotted the top five active regulons in each distinct cluster, demonstrated that the majority of cells enriched for these five TFs are multi‐HSCs and MPP1s (Figure 4D). This finding indicates that these TFs play pivotal roles in multipotency regulation.

Next, we constructed a gene regulatory network including these TFs and their direct target genes (Figure 4E). The core TF *Hoxb5* (encoding Hox‐B5) in this regulatory network has been identified as a critical TF expressed in long‐term HSCs and has been used to isolate long‐term HSCs.^26^ To verify the critical roles of *Hoxb5* and *Spi1* in HSCs, we generated triple lineage‐tracing mice for Hox‐B5, PU.1 and GATA‐1. By using these mice, we found that 82.6% of Hox‐B5^+^Lin^−^ bone marrow cells were PU.1^dim^GATA‐1^−^, and 42.6% of Hox‐B5^+^Lin^−^PU.1^dim^GATA‐1^−^ cells tended to be CD34^−^CD48^−^ (Figure S5A). In contrast, 79.3% of Hox‐B5^+^Lin^−^ bone marrow cells were Sca1^+^c‐kit^+^, and 42.4% of Hox‐B5^+^Lin^−^Sca1^+^c‐kit^+^ cells tended to be CD34^−^CD48^−^. Bone marrow transplantation assays revealed that PU.1^dim^GATA‐1^−^ but not other cells in the Hox‐B5^+^Lin^−^ population gave rise to long‐term multipotent haematopoietic reconstitution (Figure S5B).

## 4 DISCUSSION

Recent studies revealed TFs can be used to define HSCs in mouse bone marrow.^26^, ^27^, ^28^, ^29^, ^30^, ^31^, ^32^ While the debate on the heterogeneity and fate determination of HSCs is ongoing, existing studies support the notion that haematopoietic lineage‐specific TFs contribute to multiple HSC fate transformations. The scRNA‐seq data from cells in the LSK population suggest that two HSC clusters, with distinct haematopoietic potential, can be distinguished by differential expression of *Spi1* and *Gata1*. The HSC1 cluster, with moderate expression of *Spi1*, expresses the myeloid and lymphoid lineage‐specific TF genes *Egr3* and *Gata3*, which suggests the multipotency of HSC1; while the HSC2 cluster, with high expression of *Gata1*, shows up‐regulated expression of the erythroid lineage‐specific TF genes *Bmi1*, *Gfi1b*, *Gata1* and *Klf1* (Figure 1D), which indicates the erythroid‐biassed differentiation potential of HSC2. By using a lineage‐tracing mouse model, in which the expression of *Spi1* and *Gata1* is reported by the expression of GFP and mCherry respectively. *Gata1* is negatively expressed in HSC1 cluster but positively expressed in HSC2 cluster. We performed colony‐forming unit (CFU) assay in vitro and confirmed that the GATA‐1^−^ HSCs showed significantly enhanced myeloid differentiation potential compared with GATA‐1^+^ HSCs, while the GATA‐1^+^ HSCs possessed significantly enhanced erythroid‐biassed differentiation potential compared with GATA‐1^−^ HSCs (Figure S2D). By primary and secondary bone marrow transplantation assays, we have demonstrated that only the cells gated by Lin^−^PU.1^dim^GATA‐1^−^ display multipotency (Figure 3C,D). Intriguingly, analysis of the HSC populations defined by the traditional surface marker combinations CD34^−/dim^CD48^−^Lin^−^Sca1^+^EPCR^+^ (CD34^−^CD48^−^LSE), CD34^−/dim^CD48^−^Lin^−^Sca1^+^c‐kit^+^ (CD34^−^CD48^−^LSK) and CD34^−/dim^CD41^+^Lin^−^Sca1^+^c‐kit^+^ (CD34^−^CD41^+^LSK) demonstrated that the majority of the HSC population gated by CD34^−^CD48^−^LSE and CD34^−^CD48^−^LSK are PU.1^dim^GATA‐1^−^, while CD34^−^CD41^+^LSK gates HSCs including both GATA‐1^+^ and GATA‐1^−^ populations (Figure 2F–I).

By SCENIC analysis on integrated data from LPG and LSK cells, we uncovered that a regulatory network, containing five core TFs (*Zfp612*, *Foxq1*, *Hoxb5*, *Hoxa10* and *Smad6*) and five downstream co‐regulated genes (*Prdm16*, *Ski*, *Mecom*, *Nrarp* and *Efna3*), exists in multipotent HSC subsets. This network may play important roles in HSC fate determination. While *Hoxa10*, *Prdm16*, *Ski*, *Smad6* and *Efna3* have been demonstrated to be critical for HSC multipotency maintenance,^95^, ^96^, ^97^, ^98^, ^99^, ^100^ the transcription factors MECOM and Hox‐B5 have been shown to be highly expressed in HSCs of mouse bone marrow and are essential for HSC self‐renewal.^26^, ^29^, ^101^ Accordingly, we found that *Mecom* and *Hoxb5* are highly expressed in the multipotent HSC1 population refined from the integrated LPG and LSK populations (Figure S4B). Furthermore, the majority of Lin^−^Hox‐B5^+^ cells are PU.1^dim^GATA‐1^−^, and only the PU.1^dim^GATA‐1^−^ subpopulation of Lin^−^Hox‐B5^+^ cells possess long‐term haematopoietic reconstitution capacity (Figure S5A,B).

Existing studies have demonstrated that the bone morphogenetic protein (BMP) signalling pathway is activated in myeloid‐lymphoid balanced rather than myeloid‐biassed HSC populations,^102^ while the transforming growth factor beta (TGF‐β) signalling pathway is generally activated in myeloid‐biassed HSC populations.^103^ Accordingly, the core TF SMAD6 that we identified here by regulon analysis has been reported to be correlated with the BMP signalling pathway and inhibits erythropoiesis.^104^ The TF SKI, which is co‐regulated by SMAD6 and HOXA10, inhibits the TGF‐β signalling pathway.^98^

In addition, histone demethylation has been revealed to be required for haematopoietic stem cell maintenance.^105^ The TF MECOM, which is predicted to be co‐regulated by SMAD6, Hox‐B5, FOXQ1 and ZFP612, directly interacts with the histone methyltransferase SUV39H1 to form an active complex with methyltransferase activity, and the TF PRDM16, which is co‐regulated by Hox‐B5 and Hox‐A10, is an H3K9me1 methyltransferase.^106^, ^107^ These data may suggest undefined novel epigenetic and transcriptional mechanisms that regulate HSC multipotency.

In conclusion, by combining single‐cell transcriptomics with lineage tracing, we uncover that LPG defines a population of mouse bone marrow HSCs with the capacity to reconstitute multiple haematopoietic lineages. In addition, by using pySCENIC analysis of integrated data from LPG and LSK cells, we identified a novel gene regulatory network consisting of the core TFs *Zfp612*, *Foxq1*, *Hoxb5*, *Hoxa10* and *Smad6*. The detailed mechanism of how these core TFs and their co‐regulated genes coordinate to regulate HSC fate requires further investigation.

## AUTHOR CONTRIBUTIONS

Tongbiao Zhao, Jiani Cao and Haoyu Xu designed the project; Haoyu Xu, Yu Zhao, Lin Zhang, Xing Li, Weiyun Cao and Jiayi Tian performed the experiments; Fengchao Wang contributed vital mouse models; Haoyu Xu and Shaojing Tan analysed the scRNA‐seq data; Tongbiao Zhao supervised the experiments; Haoyu Xu, Jiani Cao and Tongbiao Zhao wrote and edited the manuscript. All authors reviewed the manuscript.

## CONFLICT OF INTEREST

The authors declare no competing financial interest.

## Supporting information


**Figure S1.** Detection of PU.1 and GATA‐1 expression in CLP, CMP, MEP, GMP and haematopoietic lineages using PGdKI mice. (A) Flow cytometry analysis of bone marrow progenitor cells (CLP, CMP, MEP and GMP). (B) Flow cytometry analysis of differentiated lymphoid lineages in peripheral blood (NK cells, B cells, CD4SP and CD8SP cells). (C) Flow cytometry analysis of erythroid cells (RBC) and platelets in peripheral blood. (D) Flow cytometry analysis of myeloid lineages in peripheral blood (DCs, Mφ and neutrophils). CLP: common lymphoid cells; CMP: common myeloid cells; MEP: megakaryocyte‐erythroid progenitor cells; GMP: granulocyte–macrophage progenitor cells; CD4SP: CD4 single‐positive; CD8SP: CD8 single‐positive; RBC: red blood cells; DCs: dendritic cells; Mø: macrophages.

**Figure S2.** The expression of fluorescent proteins is closely correlated with the expression of endogenous PU.1 and GATA‐1 in PGdKI mice. (A) The FACS‐sorted eGFP^−^, eGFP^dim^, and eGFP^high^ cells were stained with anti‐GFP (purple) and anti‐PU.1 (red) antibodies and detected by confocal microscopy. eGFP, auto‐fluorescence of GFP (green); DAPI, nuclei were stained with DAPI (blue). Bar = 50 μm. (B) FACS‐sorted mCherry^+^ and mCherry^−^ cells in mouse bone marrow. (C) Western blotting for GATA‐1 and β‐actin in mCherry^+^ and mCherry^−^ bone marrow cells. β‐Actin served as the loading control. (D) GATA‐1^−^ HSCs and GATA‐1^+^ HSCs from PGdKI mice were plated in Methocult for colony‐forming unit (CFU) analysis 7 days after plating, *n* = 4. Data are shown as mean ± s.e.m., *p‐value <0.05. CFU‐M: colony‐forming unit‐myeloid; BFU‐E: blast‐forming CFU‐erythroid. (E) FACS analysis showing the LSK cells in LPG cells and LPG cells in LSK cells. (F) The percentages of LSK cells in LPG cells and LPG cells in LSK cells. Data are shown as mean ± s.e.m., *n* = 3.

**Figure S3.** Expression of the indicated haematopoietic cell marker genes erythroid genes in distinct cell clusters. (A) UMAP visualization of the expression patterns of the canonical haematopoietic cell marker genes *Cd34*, *Cd48*, *Flt3*, *Dach1*, *Il7r*, *Dntt*, *Itgax*, *Itgam*, *Fcgr3*, *Fcgr2b*, *Ptprc*, *Cd22*, *Rag1*, *Ms4a1*, *Cd79a* and *Cd81* in distinct cell clusters. Each dot represents one cell. **(B)** UMAP visualization of the expression patterns of the erythroid genes *Bmi1*, *Gfi1b*, *Gata1*, *Nfe2*, *Klf1*, *Fli1*, *Vwf and Mpl* in distinct cell clusters. Each dot represents one cell.

**Figure S4.** Regulon specificity scores in each indicated haematopoietic lineage. (A) The top five regulons are labelled by red dots from each haematopoietic lineage according to their specificity scores. (B) Heatmap showing the mean expression of the top 5 regulon‐representing TF genes in each cluster. The top TF genes are labelled in the same font colour as the corresponding cluster.

**Figure S5.** The majority of cells within the Lin^−^Hox‐B5^+^ population are PU.1^dim^GATA‐1^−^. (A) FACS analysis indicates that 82.6% of Lin^−^Hox‐B5^+^ cells are Sca1^+^c‐kit^+^, and 79.3% of Lin^−^Hox‐B5^+^ cells are PU.1^dim^GATA‐1^−^. In Hox‐B5^+^LSK cells, 42.4% are CD34^−^CD48^−^ HSCs, and in Hox‐B5^+^LPG cells, 42.6% are CD34^−^CD48^−^ HSCs. (B) Bone marrow transplantation assays to assess the ability of Hox‐B5^+^ cells within the PU.1^dim^GATA‐1^−^ fraction or outside the PU.1^dim^GATA‐1^−^ fraction to reconstitute long‐term haematopoiesis at 32 weeks post‐transplantation (*n* = 6 mice for Hox‐B5^+^PU.1^dim^GATA‐1^−^ subsets; *n* = 6 mice for Hox‐B5^+^ cells but not PU.1^dim^GATA‐1^−^, finally. Shown were average values for individual recipient mice pooled from three independent experiments. Error bars denote SD).

## Data Availability

The raw data files for the scRNA sequencing data have been deposited in the Science Data Bank. All relevant data supporting the findings of this study are also available from the lead contact (tbzhao@ioz.ac.cn) upon request.
