# Supplementary figures and images for: Lin− PU.1dimGATA‐1− defines haematopoietic stem cells with long‐term multilineage reconstitution activity
Source: Cell Prolif. 2023 May 5;56(11):e13490. doi: 10.1111/cpr.13490 (PMC10623959; doi:10.1111/cpr.13490)

Supplemental Figure 1

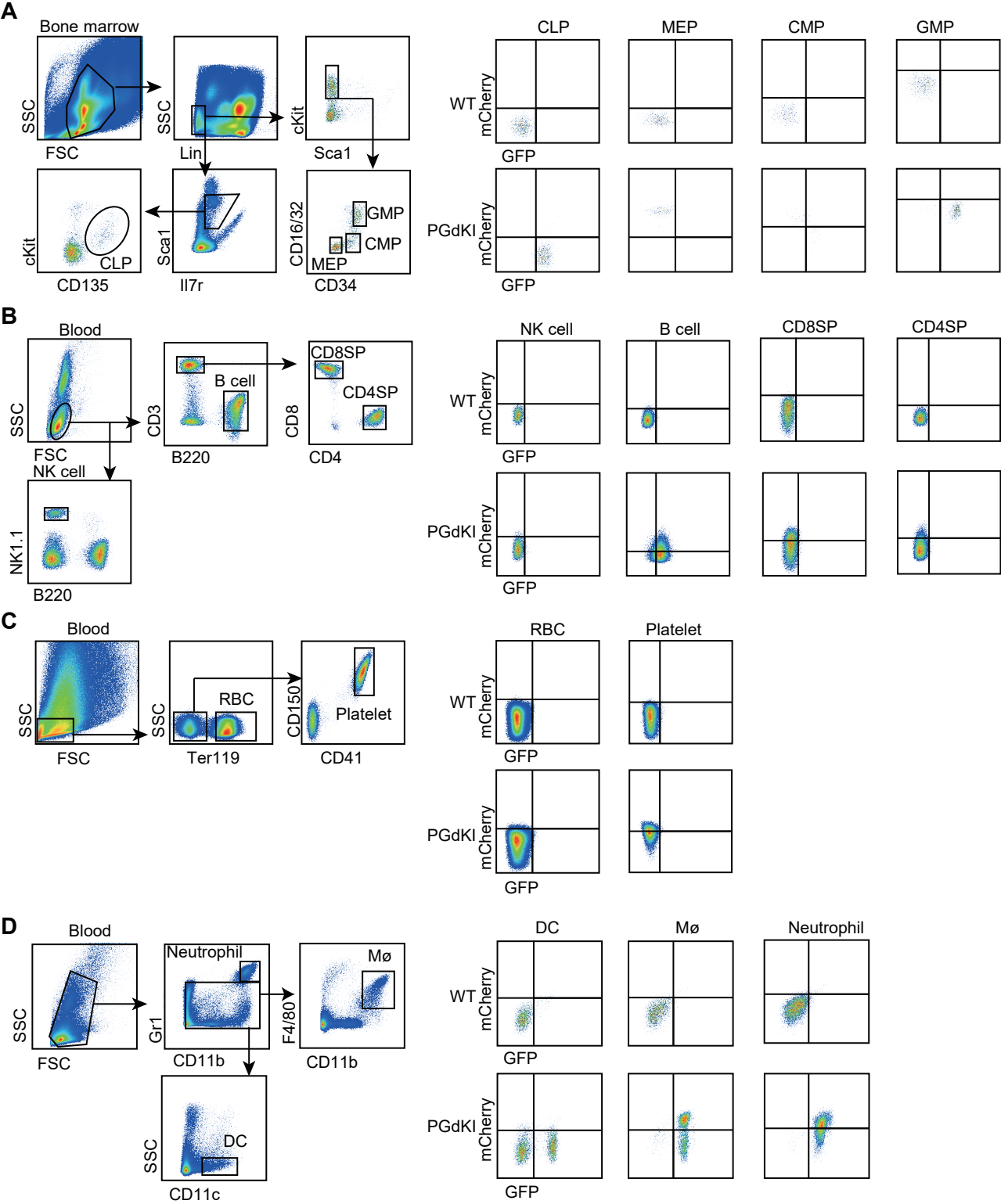

Supplement: Supplementary file 1 — Figure S1. Detection of PU.1 and GATA‐1 expression in CLP, CMP, MEP, GMP and haematopoietic lineages using PGdKI mice. (A) Flow cytometry analysis of bone marrow progenitor cells (CLP, CMP, MEP and GMP). (B) Flow cytometry analysis of differentiated lymphoid lineages in peripheral blood (NK cells, B cells, CD4SP and CD8SP cells). (C) Flow cytometry analysis of erythroid cells (RBC) and platelets in peripheral blood. (D) Flow cytometry analysis of myeloid lineages in peripheral blood (DCs, Mφ and neutrophils). CLP: common lymphoid cells; CMP: common myeloid cells; MEP: megakaryocyte‐erythroid progenitor cells; GMP: granulocyte–macrophage progenitor cells; CD4SP: CD4 single‐positive; CD8SP: CD8 single‐positive; RBC: red blood cells; DCs: dendritic cells; Mø: macrophages. [file CPR-56-e13490-s005.pdf]

## Supplemental Figure 2

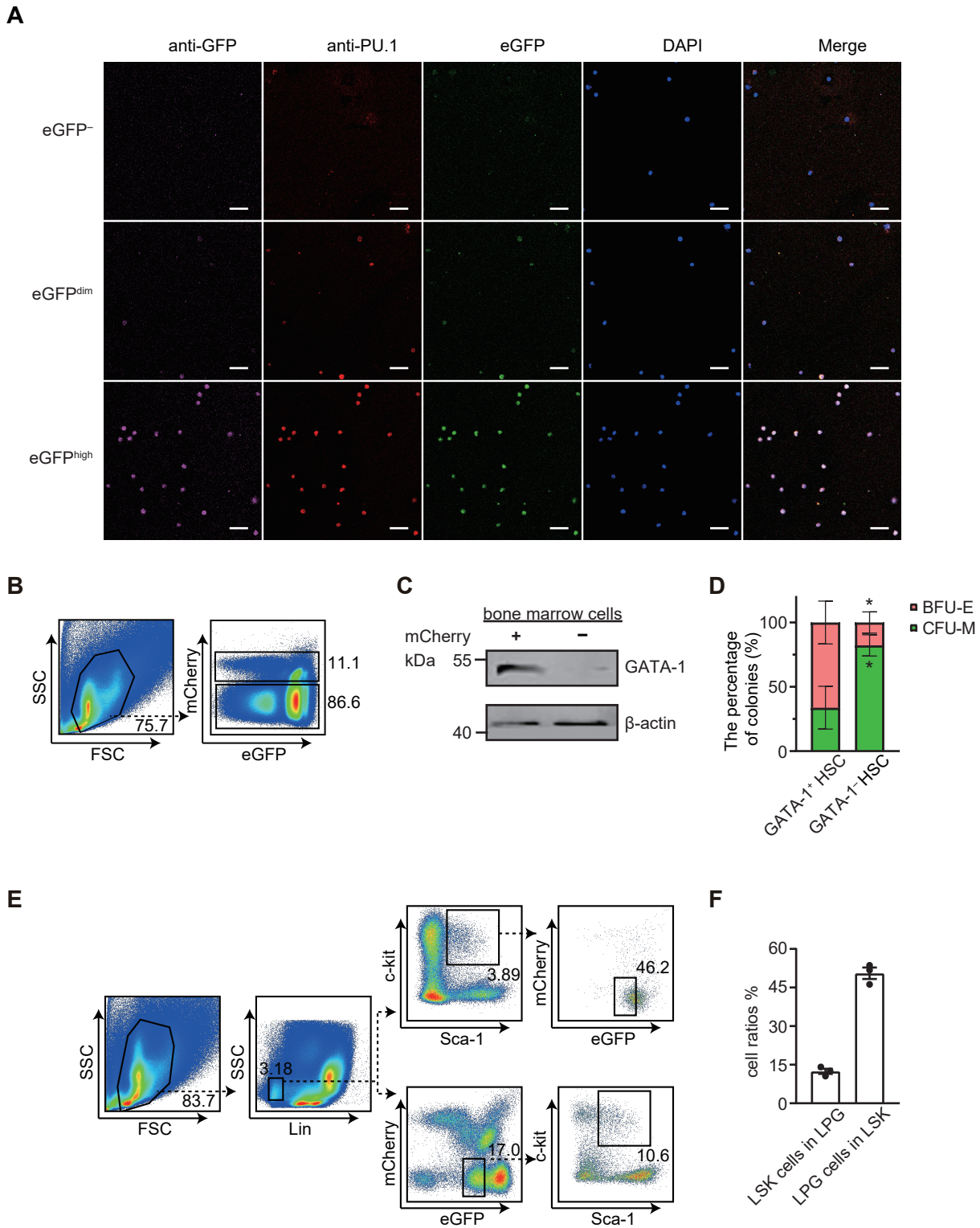

Supplement: Supplementary file 2 — Figure S2. The expression of fluorescent proteins is closely correlated with the expression of endogenous PU.1 and GATA‐1 in PGdKI mice. (A) The FACS‐sorted eGFP−, eGFPdim, and eGFPhigh cells were stained with anti‐GFP (purple) and anti‐PU.1 (red) antibodies and detected by confocal microscopy. eGFP, auto‐fluorescence of GFP (green); DAPI, nuclei were stained with DAPI (blue). Bar = 50 μm. (B) FACS‐sorted mCherry+ and mCherry− cells in mouse bone marrow. (C) Western blotting for GATA‐1 and β‐actin in mCherry+ and mCherry− bone marrow cells. β‐Actin served as the loading control. (D) GATA‐1− HSCs and GATA‐1+ HSCs from PGdKI mice were plated in Methocult for colony‐forming unit (CFU) analysis 7 days after plating, n = 4. Data are shown as mean ± s.e.m., *p‐value <0.05. CFU‐M: colony‐forming unit‐myeloid; BFU‐E: blast‐forming CFU‐erythroid. (E) FACS analysis showing the LSK cells in LPG cells and LPG cells in LSK cells. (F) The percentages of LSK cells in LPG cells and LPG cells in LSK cells. Data are shown as mean ± s.e.m., n = 3. [file CPR-56-e13490-s002.pdf]

## Supplemental Figure 3

**A**

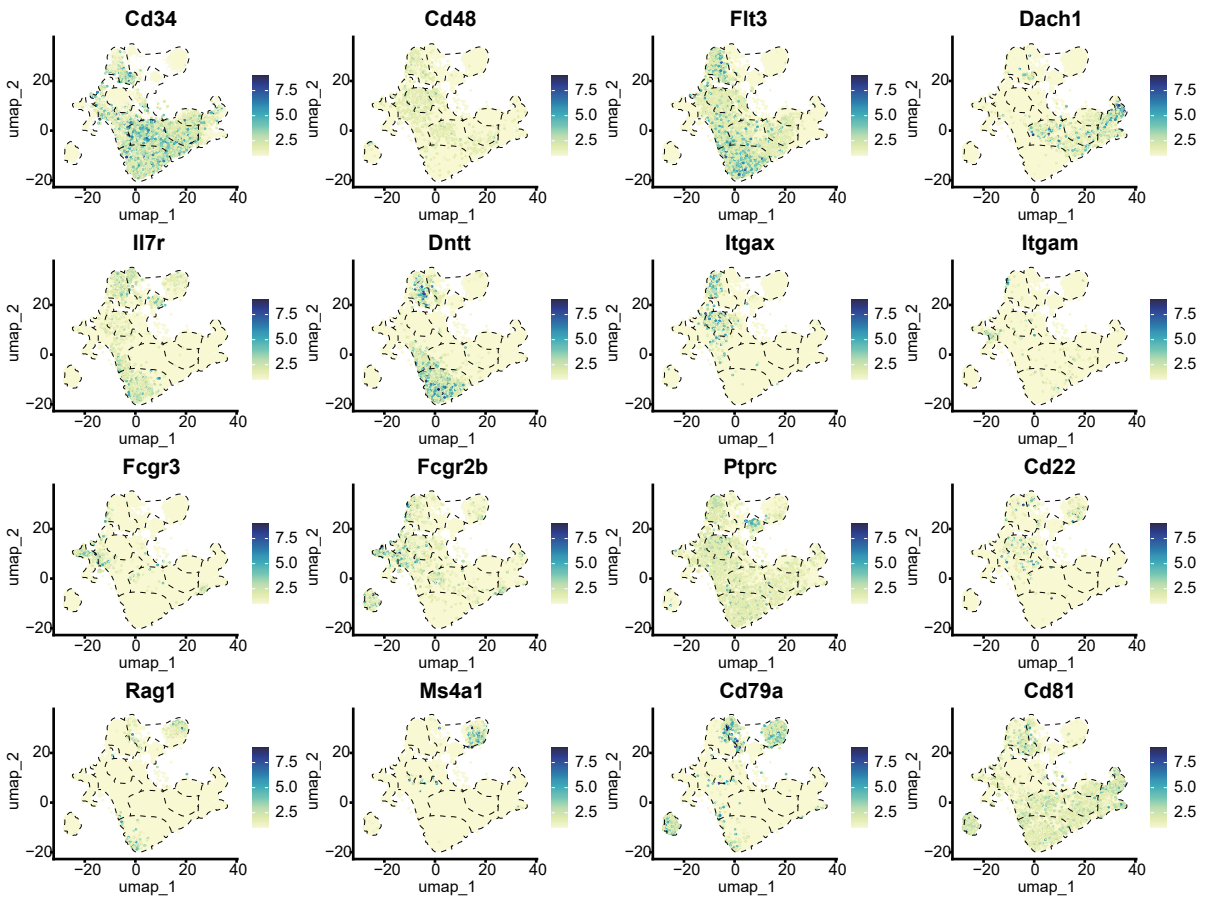

**B**

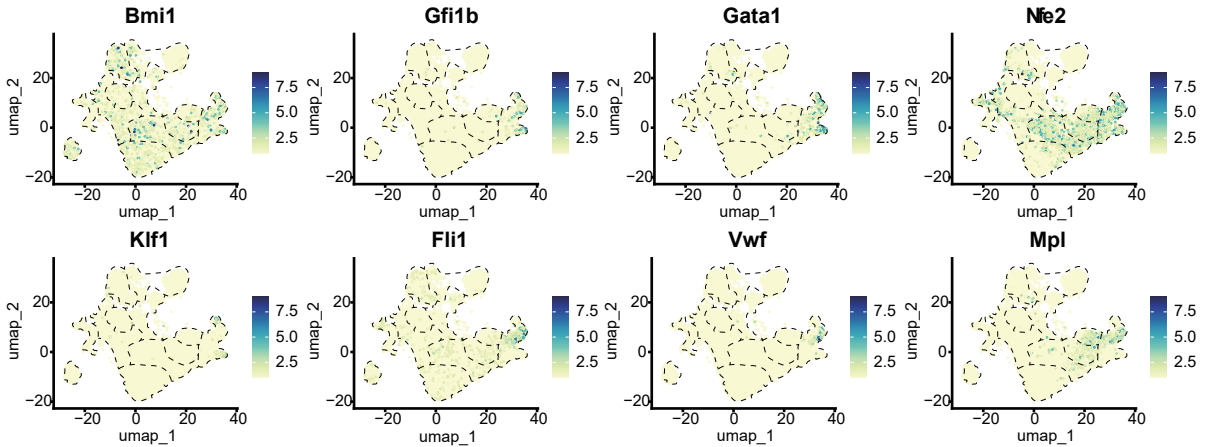

Supplement: Supplementary file 3 — Figure S3. Expression of the indicated haematopoietic cell marker genes erythroid genes in distinct cell clusters. (A) UMAP visualization of the expression patterns of the canonical haematopoietic cell marker genes Cd34, Cd48, Flt3, Dach1, Il7r, Dntt, Itgax, Itgam, Fcgr3, Fcgr2b, Ptprc, Cd22, Rag1, Ms4a1, Cd79a and Cd81 in distinct cell clusters. Each dot represents one cell. (B) UMAP visualization of the expression patterns of the erythroid genes Bmi1, Gfi1b, Gata1, Nfe2, Klf1, Fli1, Vwf and Mpl in distinct cell clusters. Each dot represents one cell. [file CPR-56-e13490-s004.pdf]

## Supplemental Figure 4

**A**

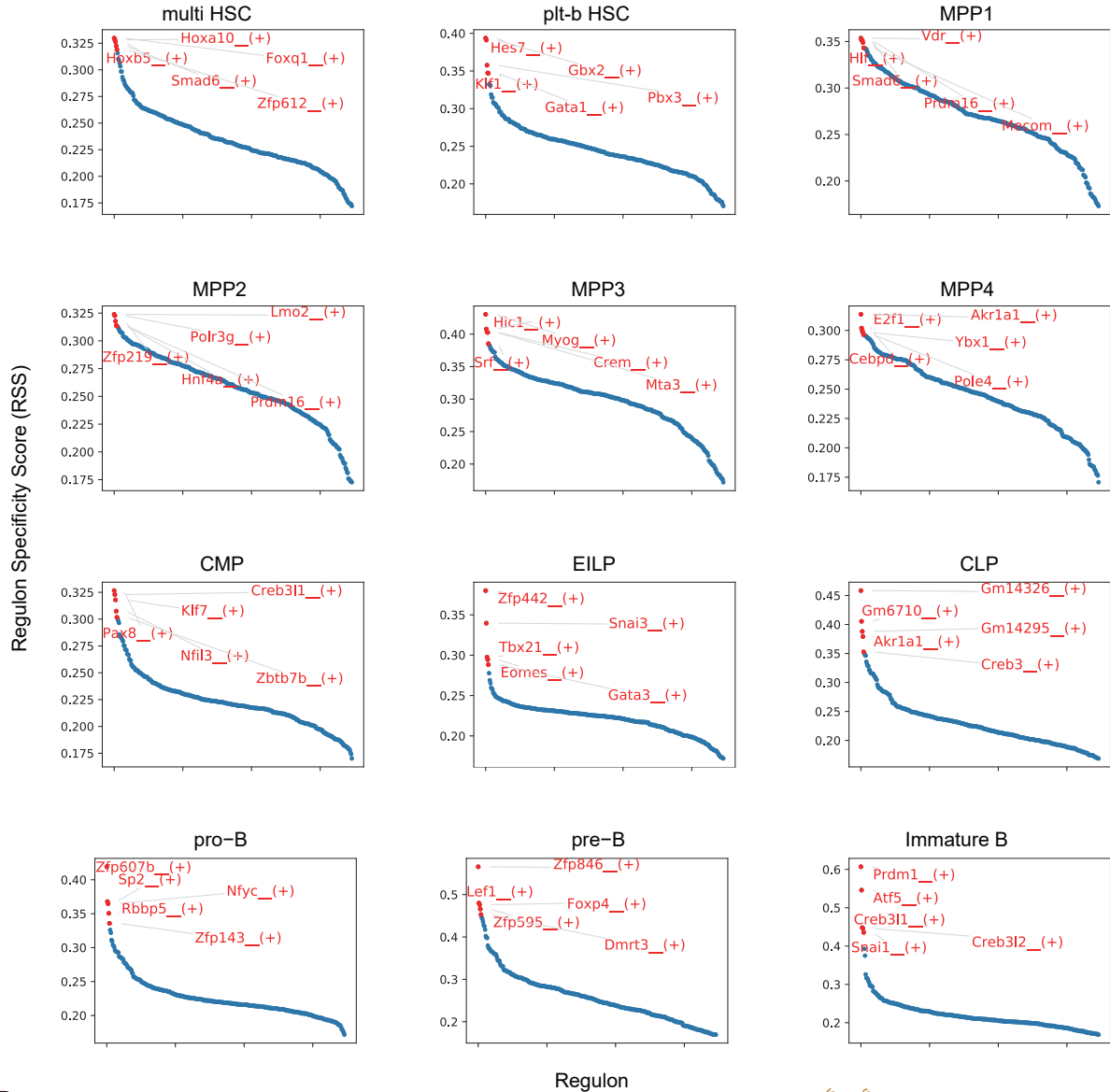

**B**

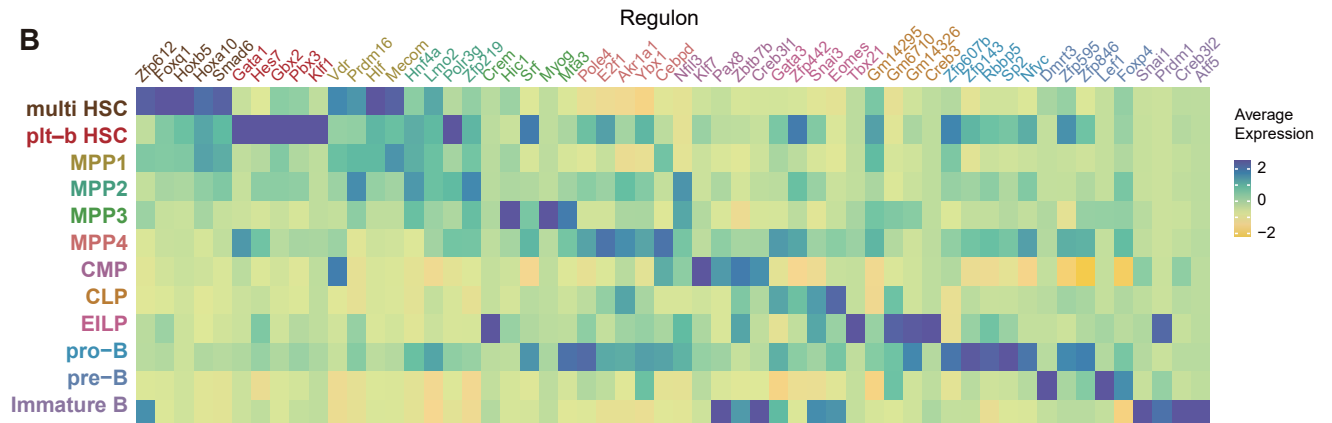

Supplement: Supplementary file 4 — Figure S4. Regulon specificity scores in each indicated haematopoietic lineage. (A) The top five regulons are labelled by red dots from each haematopoietic lineage according to their specificity scores. (B) Heatmap showing the mean expression of the top 5 regulon‐representing TF genes in each cluster. The top TF genes are labelled in the same font colour as the corresponding cluster. [file CPR-56-e13490-s001.pdf]

**Supplemental Figure 5**

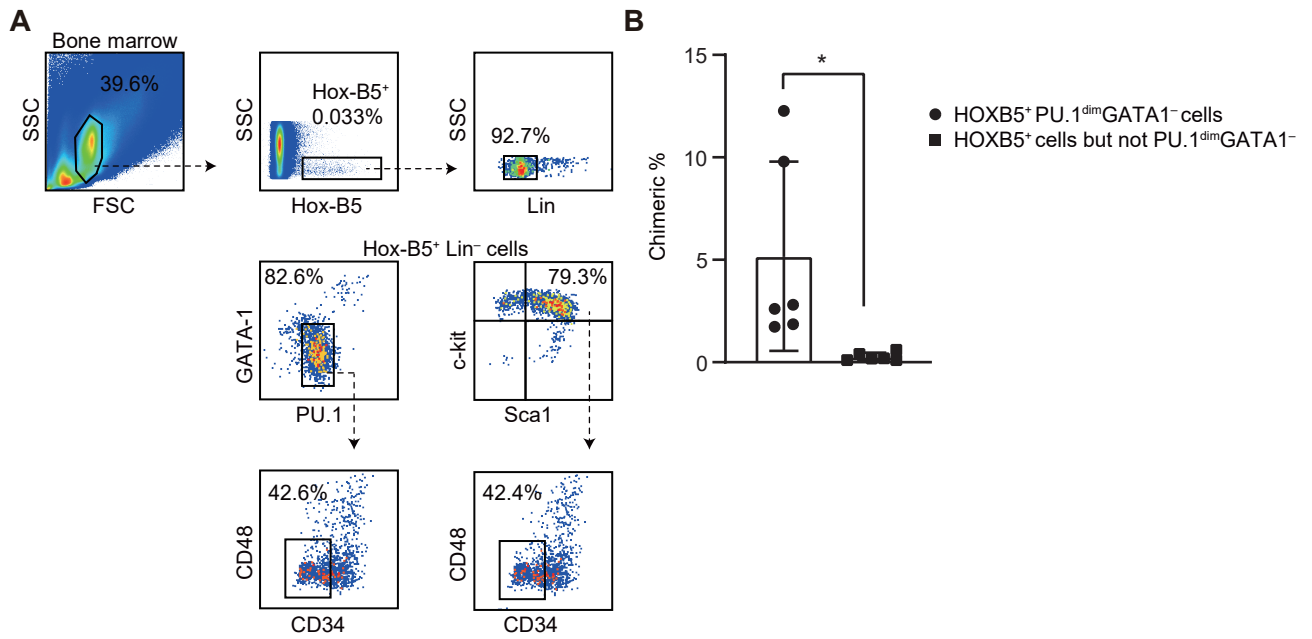

Supplement: Supplementary file 5 — Figure S5. The majority of cells within the Lin−Hox‐B5+ population are PU.1dimGATA‐1−. (A) FACS analysis indicates that 82.6% of Lin−Hox‐B5+ cells are Sca1+c‐kit+, and 79.3% of Lin−Hox‐B5+ cells are PU.1dimGATA‐1−. In Hox‐B5+LSK cells, 42.4% are CD34−CD48− HSCs, and in Hox‐B5+LPG cells, 42.6% are CD34−CD48− HSCs. (B) Bone marrow transplantation assays to assess the ability of Hox‐B5+ cells within the PU.1dimGATA‐1− fraction or outside the PU.1dimGATA‐1− fraction to reconstitute long‐term haematopoiesis at 32 weeks post‐transplantation (n = 6 mice for Hox‐B5+PU.1dimGATA‐1− subsets; n = 6 mice for Hox‐B5+ cells but not PU.1dimGATA‐1−, finally. Shown were average values for individual recipient mice pooled from three independent experiments. Error bars denote SD). [file CPR-56-e13490-s003.pdf]
